# Synthetic Cannabinoid and Mitragynine Exposure of Law Enforcement Agents During the Raid of an Illegal Laboratory — Nevada, 2014

**DOI:** 10.15585/mmwr.mm6647a3

**Published:** 2017-12-01

**Authors:** Loren Tapp, Jessica G. Ramsey, Anita Wen, Roy Gerona

**Affiliations:** ^1^Division of Surveillance, Hazard Evaluations, and Field Studies, National Institute for Occupational Safety and Health, CDC; ^2^Clinical Toxicology and Environmental Biomonitoring Laboratory, University of California, San Francisco.

Synthetic cannabinoids (SCs), commonly known by the street name “Spice,” are designer drugs of abuse that mimic the psychoactive effects of marijuana. Intentional SC use has resulted in multiple toxicities (*1*,*2*), but little is known about occupational SC exposure. After a federal agency’s law enforcement personnel in Nevada reported irritability and feeling “high” after raiding illegal SC laboratories and processing seized SCs, a request for a health hazard evaluation was made by the agency to CDC’s National Institute for Occupational Safety and Health (NIOSH) in 2014 to evaluate agents’ occupational SC exposures. After making the request for a health hazard evaluation, federal agents conducted a raid of an illegal SC laboratory, with assistance from local law enforcement and Drug Enforcement Administration (DEA) personnel and with NIOSH investigators observing from a distance. After the raid, agents collected and processed material evidence. NIOSH investigators tested agents’ urine for SC levels before and after the raid and measured SCs in the air and on surfaces after the raid. DEA determined that AB-PINACA (an SC compound) and mitragynine (a plant material with opium-like effects, also known as “kratom”) were present in the illegal laboratory. AB-PINACA, its metabolites, and mitragynine were not detected in agents’ urine before the raid; however, one or more of these substances was found in the urine of six of nine agents after the raid and processing of the SC evidence. AB-PINACA was detected in one surface wipe sample from the SC laboratory; none was detected in the air in the laboratory or in the offices of the law enforcement agency where the materials were processed after the raid. No policies were in place regarding work practices and use of personal protective equipment (PPE) during raids and evidence processing. To protect agents from SC exposures, NIOSH recommended that the agency require agents to wear a minimum level of PPE (e.g., protective gloves and disposable clothing) and undergo training in PPE and in handling and storing of contaminated evidence from SC laboratory raids. Showers and locker rooms also need to be provided so that agents can reduce contamination and prevent take-home exposure.

Shortly after NIOSH received the request for a health hazard evaluation, an opportunity arose for federal agents to plan a raid of an illegal SC laboratory with a 1-week lead time. A health hazard evaluation site visit was immediately planned in response to the agency’s concerns. Four NIOSH investigators arrived at the agency’s office 1 day before the raid and participated in a briefing to discuss roles and responsibilities. Agents’ activities, work and hygiene practices, and PPE use were observed, and sampling was conducted before and after the raid. Eighteen personal breathing zone air samples and seven area air samples (from the SC laboratory and the agents’ office) were collected over 2 days. Seventeen surface wipe samples were collected from the SC laboratory and the agents’ office. Air and surface wipe samples were analyzed for AB-PINACA and mitragynine using gas chromatography–mass spectrometry at Bureau Veritas North America, Novi, Michigan. No validated method exists for quantitative air sampling of SC, so a modified DEA method using gas chromatography–mass spectrometry and methanol extraction was used. The results obtained should be considered semiquantitative and the reported minimum detectable and minimum quantifiable concentrations should be considered estimates. The ventilation system of the office was evaluated by measuring airflow using a ventilation flow hood.

Nine agents completed a questionnaire and, after providing informed consent, submitted urine samples for SC analyses. The questionnaire asked about demographics and work, medical, and smoking history; alcohol use; and pertinent health symptoms ever experienced when handling SC evidence. Each agent provided five urine specimens over 3 consecutive days. The first specimen (baseline) was collected on designated day 1 (the day before the raid); the second was collected at the end of the shift on day 2 (after the raid and evidence collection). The third was collected at bedtime on day 2; the fourth was collected before beginning the shift on day 3, and the fifth was collected at the end of the shift on day 3 (after sorting evidence in the office) (Table). Liquid chromatography–tandem mass spectrometry testing was performed at the Clinical Toxicology and Environmental Biomonitoring Laboratory, University of California, San Francisco, to measure the amount of AB-PINACA, two AB-PINACA metabolites (AB-PINACA-[4-hydroxypentyl] metabolite and AB-PINACA N-pentanoic acid), and mitragynine in all urine specimens.

**TABLE:** Presence* of cannabinoid metabolites and mitragynine in the urine of nine law enforcement agents, detected by liquid chromatography–tandem mass spectrometry before and after a raid on an illegal synthetic cannabinoid laboratory — Nevada, 2014

Agent	Day 1 (pre-raid) baseline	Day 2 (raid) post-shift	Day 2 (raid) at bedtime	Day 3 (post-raid) morning	Day 3 (post-raid) post-shift
1	None	None	None	Mitragynine	Mitragynine
2	None	AB-PINACA, AB-PINACA OH, AB-PINACA pent, mitragynine	AB-PINACA, AB-PINACA OH, AB-PINACA pent, mitragynine	AB-PINACA pent, mitragynine	AB-PINACA pent, mitragynine
3	None	None	AB-PINACA pent, mitragynine	None	None
4	None	None	None	None	None
5	None	None	None	None	None
6	None	AB-PINACA, AB-PINACA pent, mitragynine	AB-PINACA pent, mitragynine	AB-PINACA pent, mitragynine	AB-PINACA pent, mitragynine
7	None	Mitragynine	Mitragynine	Mitragynine	Mitragynine
8	None	None	None	None	None
9	None	AB-PINACA, mitragynine	Mitragynine	Mitragynine	Mitragynine

**Abbreviations:** AB-PINACA OH = AB-PINACA N-(4-hydroxypentyl) metabolite; AB-PINACA pent = AB-PINACA N-pentanoic acid metabolite.

*Any level above the lower limit of quantification (LLOQ) for the substance. The LLOQ for AB-PINACA, AB-PINACA OH, and AB-PINACA pent is 0.10 ng/mL; the LLOQ for mitragynine is 1.0 ng/mL.

NIOSH investigators observed agents’ practices during the raid and evidence collection and processing. During the raid (day 2), the agents collected suspect plant material, sealed packets containing SC and mitragynine, baking sheets, and paper records. The evidence was placed in plastic or paper bags and stored in a locked evidence room located in the agency offices. The next day (day 3), the agents took evidence bags from the evidence room to a carpeted conference room, an adjacent equipment room with tiled flooring, and individual offices for sorting and cataloging.

During the raid, some agents voluntarily wore disposable protective clothing, and one wore a filtering face-piece respirator during part of the raid. No agents wore disposable shoe coverings. The use of latex or nitrile gloves during the raid and evidence processing was inconsistent. Some agents entered the office after the raid without changing out of the clothing (either personal or disposable) worn during the raid. Agents were not provided with a designated locker room or showers. Agents were observed to be eating and drinking while handling and cataloging the evidence. The evidence storage and processing rooms were connected to the ventilation system that also supplied the other office areas. The ventilation system, the layout of the offices, and the agents’ work practices were not designed to contain or control forensic hazards.

All nine participating agents were men, and their average age was 45 years (range = 35–60 years). The average time in their current job was 9 years (range = 4–19 years). Some agents reported tobacco or alcohol use, exposure to solvents outside of work, chronic medical conditions, and prescription medication use. All reported previously handling SC material evidence from previous drug raids. Approximately half of the nine agents reported ever having cough, eye irritation, throat irritation, and dizziness or lightheadedness (based on recall) from previous activities handling SC (Figure). Four agents reported feeling “high” when handling SC and three of these four also reported irritability, difficulty remembering things, and difficulty concentrating, which are frequently experienced by persons with intentional SC exposure.

**FIGURE:**
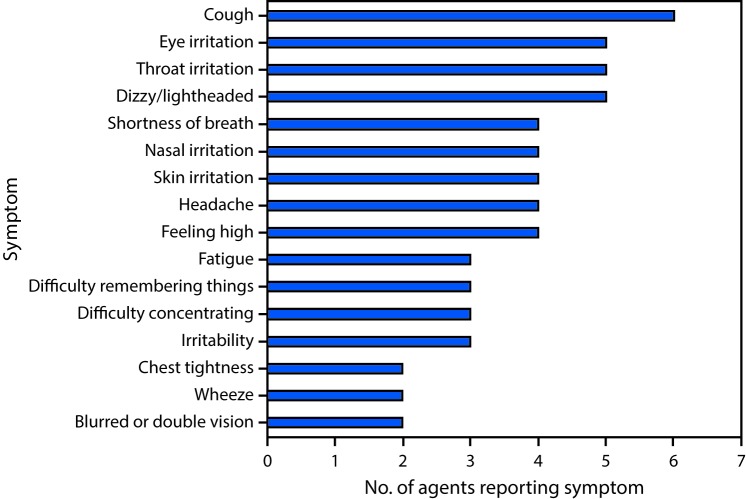
Symptoms reported by nine law enforcement agents involved in the raid of an illegal synthetic cannabinoid laboratory while ever handling* synthetic cannabinoids — Nevada, 2014 **Source:** Adapted from Ramsey JG, Tapp L, Burr G. Evaluation of law enforcement agents’ potential exposures during a raid of a clandestine “spice” lab. HHE report no. 2014-0039-3246. US Department of Health and Human Services, CDC, National Institute for Occupational Safety and Health; 2014. https://www.cdc.gov/niosh/hhe/reports/pdfs/2014-0039-3246.pdf. * Refers to previous raids of illegal "spice" laboratories and evidence handling, not to the raid and evidence handling during this investigation.

All nine agents reported handling or being in the same room with the SC compound during the NIOSH evaluation. Two agents reported that they always wore gloves, five said they sometimes or usually wore gloves, and two said they did not wear gloves when handling SC during this evaluation. Six agents reported that they sometimes wore respirators during their job, including dust masks, filtering face-piece respirators, or half-mask elastomeric respirators (air-purifying respirators with replaceable filters, cartridges, or canisters).

No AB-PINACA or mitragynine was found in any of the personal air or area air samples. The estimated minimum detectable concentrations for AB-PINACA and mitragynine were based on laboratory measurements and ranged from 0.03 to 0.1 mg/m^3^ and from 0.02 to 0.1 mg/m^3^, respectively. Only one wipe sample, taken from a baking sheet in the SC laboratory where treated plant material was dried, had a detectable level of AB-PINACA. No wipe samples had detectable levels of mitragynine.

Liquid chromatography–tandem mass spectrometry testing did not detect AB-PINACA, its metabolites, or mitragynine in any agent’s baseline urine specimen (Table). After the raid, however, four agents had detectable AB-PINACA or its metabolites in their urine, and six agents had mitragynine in their urine. The agent with the highest concentrations of SC metabolites was observed handling most of the evidence; this agent reported not wearing gloves. Five agents with positive urine results handled evidence during the raid and reported variable glove use, ranging from none to always; one of the five was a smoker who reported usually wearing gloves and could have had hand-to-mouth exposure. Among the three agents with negative urine testing results, two did not handle evidence during or after the raid; the third agent participated in both activities and reported some glove use.

## Discussion

Acute SC poisonings in the United States appear to be increasing, and several recent outbreaks among users have been reported (*1*,*3*). DEA has banned several SCs, but SC manufacturers continually modify these compounds to avoid illegal status, packaging them as “herbal incense” and labeling them “not for human consumption” (*4*). SCs have effects that are similar to, but often much more potent than, those of delta-9-tetrahydrocannabinol, the psychoactive compound found in marijuana (*1*,*4*,*5*). Symptoms of SC toxicity vary by exposure route, type of SC, and dose (*5*). Reported signs and symptoms of abuse include anxiety, agitation, acute psychosis, hallucinations, seizures, tachycardia, hypertension, hypokalemia, nausea, and vomiting (*1*,*2*,*4*,*5*). More severe health effects of SC abuse include respiratory depression requiring endotracheal intubation and mechanical ventilation, acute kidney injury, hyperthermia and rhabdomyolysis, and acute myocardial infarction (*2*,*4*,*5*). Few SCs are identified by routine drug screening tests (*4*).

Mitragynine is derived from the *Mitragyna speciosa* plant. It has been used for its stimulant effects (to enhance physical effort and endurance), opium-like effects (pain relief and sedation), and modulation of opiate withdrawal, but it appears to have addictive properties (*6*). Mitragynine preparations are accessible in some “smoke shops” and via the Internet (*7*).

Law enforcement agents are involved in raiding SC manufacturing laboratories and processing seized SC evidence, but little is known about their occupational exposure to SCs. A recent case series reported occupational skin exposure to SC oil resulted in transdermal SC poisoning in three customs inspectors (*8*). A previous study found that law enforcement agents participating in methamphetamine laboratory investigations had an increased number of respiratory symptoms, even with the use of respiratory protection (*9*). The levels of AB-PINACA and its metabolites found in agents’ urine were much lower than the levels reported in patients evaluated in emergency departments after intentional use (*10*); however, the levels at which health effects might occur is unknown, and no biologic exposure limits or reference ranges exist for these compounds in the occupational setting.

AB-PINACA and mitragynine were detected in urine, but not in air, suggesting that dermal absorption or ingestion from dermal contamination might be important routes of exposure. However, these compounds might also be present in air and levels might vary substantially among manufacturing laboratories.

The findings in this report are subject to at least three limitations. First, methods have not been validated for air and surface SC sampling and analyses and, therefore, levels of contamination might have been underestimated. Second, agents’ reported symptoms could not be compared with urine levels of cannabinoid metabolites and mitragynine because the symptom survey asked about historical symptoms when handling SC, not symptoms experienced during the evaluation. Finally, some agents reported tobacco or alcohol use, exposure to solvents outside of work, chronic medical conditions, and prescription medication use that might have contributed to their reported symptoms.

Law enforcement agents are at risk for dermal and ingestion exposures to SCs and other contaminants when collecting evidence in the field and processing evidence in the office; the risk for inhalation exposure is not well understood. A final report issued to the agency included the following recommendations to reduce personal exposures to potential contaminants during raids and while handling evidence: 1) properly design the forensic facility, 2) train agents on good hygiene practices, including prohibiting eating, drinking, and smoking while processing evidence, and 3) require a minimum level of PPE (e.g., disposable clothing and protective gloves) to avoid skin contact or inadvertent ingestion. Other occupations, such as housekeeping staff members in the offices, also might be at risk for exposure. Additional research exploring air exposures from SCs, take-home exposures, laboratory methods, and preventive measures is needed. The related health hazard evaluation was published online by NIOSH in March 2016 (https://www.cdc.gov/niosh/hhe/reports/pdfs/2014-0039-3246.pdf).

**Summary**

**What is already known about this topic?**

Some persons who have inhaled or ingested synthetic cannabinoids (SCs) have had severe health effects. New SCs continue to be manufactured despite Drug Enforcement Administration efforts. Law enforcement personnel have experienced symptoms during methamphetamine laboratory investigations, but little is known about their symptoms during SC enforcement activities.

**What is added by this report?**

This is the first reported investigation of occupational SC exposure. SCs or their urinary metabolites or mitragynine were detected in the urine of six of nine law enforcement agents after they were involved in raiding an illegal SC manufacturing facility and collecting, processing, and cataloging SC evidence. No policies were in place regarding the appropriate handling of evidence, such as requirements for gloves and protective clothing, or on prohibiting food and drink in evidence processing areas. Shower and locker areas were not provided for agents to reduce contamination and prevent take-home exposure. The layout and ventilation of the agency’s office did not contain or control potential hazards from the receiving, processing, and storing of evidence.

**What are the implications for public health practice?**

Law enforcement agents are exposed to material containing SCs during raids of illegal SC laboratories, and when collecting, processing, and cataloging SC evidence. A properly designed forensic facility and good hygiene practices can reduce personal exposures to potential contaminants during law enforcement raids and while handling evidence. A minimum level of personal protective equipment is needed, in addition to prohibiting eating, drinking, and smoking while processing SC evidence.
