# *Erratum*: Vol. 66, No. 47

**DOI:** 10.15585/mmwr.mm6648a6

**Published:** 2017-12-08

In the report “Synthetic Cannabinoid and Mitragynine Exposure of Law Enforcement Agents During the Raid of an Illegal Laboratory — Nevada, 2014,” on page 1292, the following footnote should have been included with the Table: “*Any level above the lower limit of quantification (LLOQ) for the substance. The LLOQ for AB-PINACA, AB-PINACA OH, and AB-PINACA pent is 0.10 ng/mL; the LLOQ for mitragynine is 1.0 ng/mL.” The online version is correct.

